# Transmission dynamics of the ongoing chikungunya outbreak in Central Italy: from coastal areas to the metropolitan city of Rome, summer 2017

**DOI:** 10.2807/1560-7917.ES.2017.22.44.17-00685

**Published:** 2017-11-02

**Authors:** Mattia Manica, Giorgio Guzzetta, Piero Poletti, Federico Filipponi, Angelo Solimini, Beniamino Caputo, Alessandra della Torre, Roberto Rosà, Stefano Merler

**Affiliations:** 1 Dipartimento di Biodiversità ed Ecologia Molecolare/Centro Ricerca e Innovazione, Fondazione Edmund Mach, San Michele all’Adige, Italy; 2 Dipartimento di Sanitá Pubblica e Malattie Infettive, Sapienza University of Rome, Laboratory affiliated to Istituto Pasteur Italia – Fondazione Cenci Bolognetti; 3 These authors contributed equally to the work; 4 Center for Information Technology, Fondazione Bruno Kessler, Trento, Italy

**Keywords:** vector-borne infections, modelling, outbreaks, chikungunya, chikungunya virus

## Abstract

A large chikungunya outbreak is ongoing in Italy, with a main cluster in the Anzio coastal municipality. With preliminary epidemiological data, and a transmission model using mosquito abundance and biting rates, we estimated the basic reproduction number R_0_ at 2.07 (95% credible interval: 1.47–2.59) and the first case importation between 21 May and 18 June 2017. Outbreak risk was higher in coastal/rural sites than urban ones. Novel transmission foci could occur up to mid-November.

On 7 September 2017, Italian public health authorities reported three autochthonous cases of chikungunya in Anzio, a coastal city 50 km south of Rome, located in the Lazio region [1]. However, the symptom onset for the first cases was retrospectively considered to have occurred between 26 and 27 June. The outbreak continued spreading in the Lazio region with the number of notified cases reaching 297 (of which 170 were confirmed) on 13 October. Although most cases were reported from Anzio, a distinct cluster of transmission was also detected in the metropolitan area of Rome [2]. The index case has not been identified, but the mosquito vector implicated in the chikungunya virus (CHIKV) transmission was confirmed to be *Aedes* *albopictus*, as was the case in a previous Italian CHIKV outbreak, which occurred in the region of Emilia Romagna in 2007 [1]. In the same period than the Lazio outbreak in 2017, a further outbreak was detected in Guardavalle Marina, a small coastal town in the Calabria region [2], 600 km south of Anzio, with 54 additional notified cases (nine confirmed). It is still unknown whether the Guardavalle outbreak is epidemiologically linked to the epidemic occurring in Lazio. Here, we provide a quantitative characterisation of the ongoing outbreak, using available epidemiological data [2] and a transmission dynamics model [3-5] informed with data on mosquito abundance [6] and biting rate on humans [7] from previous collections in 18 sites within Lazio region.

## Reproduction numbers from epidemiological data

**Figure 1:**
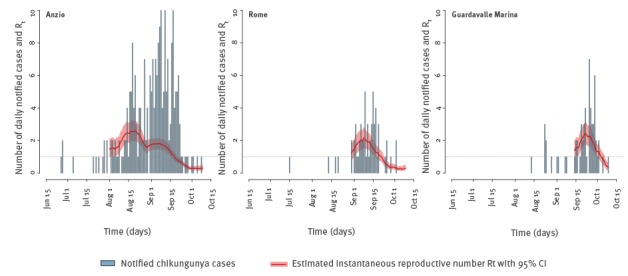
Time series of notified chikungunya cases with estimates of the instantaneous reproductive number R_t_ over time, averaged over a moving window of 14 days, Anzio, Rome and Guardavalle Marina, Italy, 2017

The instantaneous reproduction number R_t_ [8] was estimated from the time series of notified cases in Anzio, Rome and Guardavalle Marina under the assumption of gamma distributed generation time (shape = 4.67; scale = 3; mean = 4 days) [9] (Figure 1). By averaging R_t_ over the first 3 weeks of August (initial period of exponential growth), we estimated the basic reproduction number R_0_ for Anzio at 2.07 (95% credible interval (CI): 1.47–2.59), a value slightly lower than that estimated for the 2007 outbreak in Emilia Romagna (i.e. R_0_ = 3.3; 95% CI: 1.8–6.0) [3]. The decrease in R_t_ corresponded with the first date of reactive vector control interventions, namely 7 September [10]. The robustness of this estimate was confirmed by computing the basic reproduction number from the exponential growth rate [11] yielding a very similar result (R_0_ = 1.88; 95% CI: 1.55–2.27). The hypothesis of sub-exponential growth in Anzio was subsequently ruled out [12]. For Rome and Guardavalle Marina, the number of cases was too small to compute a reliable estimate of R_0_; however, peak values of R_t_ for these two outbreaks were smaller compared with the Anzio outbreak (Figure 1).

CI: credible interval; R_t_: instantaneous reproduction number.

R_t_ was estimated by Markov chain Monte Carlo applied to the Poisson likelihood associated to the renewal equation$\;C\left(t\right)={Pois(R}_{t}\sum_{s=1}^{t}T_{g}\left(s\right)C(t-s))$ [8], where *C(t)* is the number of new cases at time *t* and *T_g_* is the generation time distribution [9].

## Mosquito abundance

**Figure 2:**
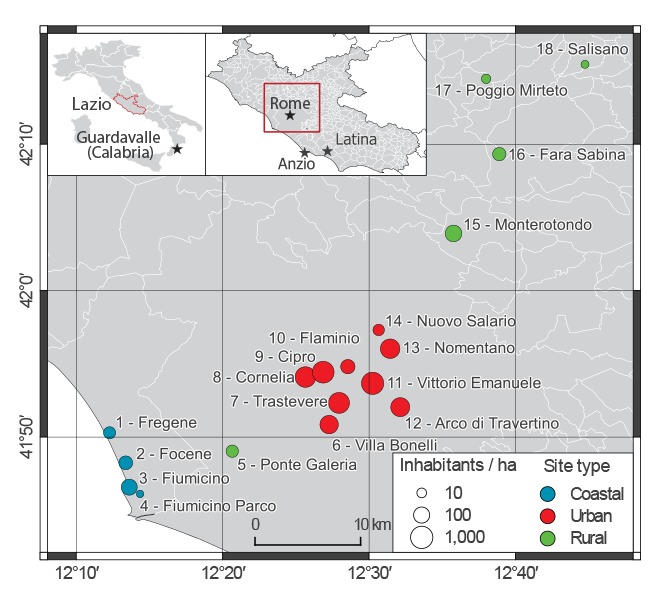
Location within the Lazio region of sites from which mosquito sampling in 2012 provided data for estimation of mosquito abundance in 2017, Italy (n = 18 sites)

We calibrated a mosquito population model [4] to *Ae.* *albopictus* capture data obtained at several time points throughout the period July to November 2012 from 18 sites along a 70 km-transect from the Lazio coast (four sites) to rural inland areas (5 sites), and encompassing the metropolitan area of Rome (nine sites) [6] (Figure 2). Coastal sites have a human density (5–50 inhabitants/ha) close to that of Anzio (roughly 30 inhabitants/ha, increasing during summer months due to touristic influx) and similar eco-climatic conditions, and were therefore considered representative for the analysis of the main outbreak; urban sites (with human density up to 267 inhabitants/ha) were considered representative for the Rome outbreak. The model takes as input daily temperature records obtained from the closest weather station to each sampling site [13]. 

Stars represent locations with ongoing outbreaks in 2017 in Italy.

**Figure 3:**
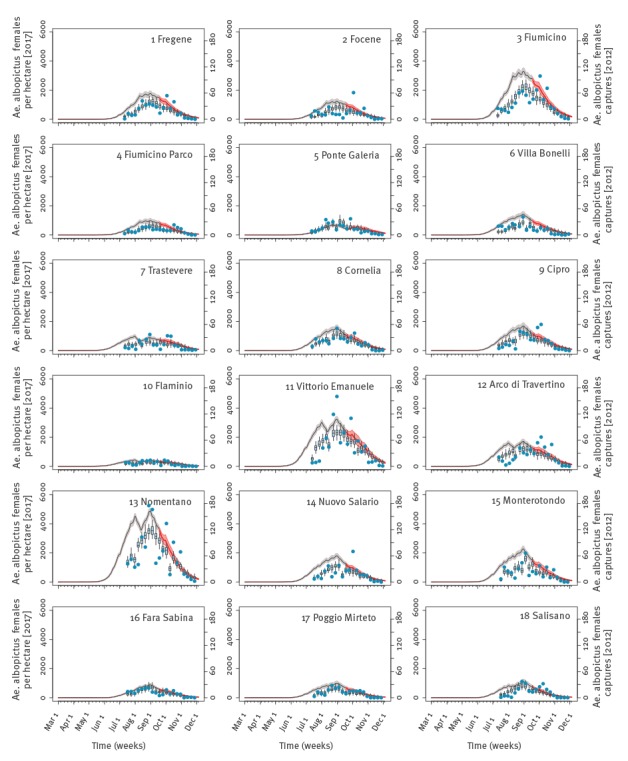
Number of *Aedes* *albopictus* adult females per hectare over time, as estimated in the absence of interventions for 2017 in the 18 mosquito sampling sites, Lazio region, Italy

The calibrated model was re-run with 2017 temperatures to estimate the mosquito abundance during the ongoing outbreak (Figure 3). Human landing capture experiments performed in 2014 within a highly *Ae.* *albopictus* infested area in Rome [7] were used to estimate the mosquito biting rate [14]. Remarkably, the biting rate was found to be nearly constant over the season and its value (range: 0.08–0.1, as shown in the Table) complies with the 0.09 (95%CI: 0.05–0.16) estimate from the 2007 CHIKV outbreak [3,14].

For each study site, the abundance of *Aedes* *albopictus* adult females per hectare in 2017 is presented over the March to December period (line: mean number; shaded area: 95% credible interval); the grey colour is used to depict estimates based on recorded temperatures [13], while red is used for estimates from predicted temperatures based on previously observed trends (scale on the left).

In addition, for each site, the observed (blue dots) and estimated (boxplots) total number of capture female adults during 2012, are shown from March to December (scale on the right). Boxplots represent 2.5%, 25%, 75%, and 97.5% quantile and mean of model estimates.

**Table:** Epidemiological parameters used in the estimation of transmission in an outbreak of chikungunya in Central Italy, 2017

Parameter	Unit	Distribution	Min and max ^a^ parameter value	Reference
Date of imported infection	Date	Uniform	1 May; 15 Nov	NA
Mosquito biting rate	Bites/mosquito/day	Uniform	0.08; 0.10	Own estimate from [7]
Probability of vector-to-human transmission per bite	%	Uniform	14; 84	[21]
Probability of human-to-vector transmission per bite	%	Uniform	75; 90	[22]
Extrinsic incubation period	Days	Uniform	2; 3	[23]
Intrinsic incubation period	Days	Uniform	1; 12	[24]
Human infectious period	Days	Uniform	2; 7	[24]
Probability of developing symptoms	%	Uniform	65; 93	[25]
Probability of being detected	%	Uniform	44; 80	[25]
Delay between symptom onset and detection	Days	Gamma	Scale: 8.53; shape: 1.725	Own estimate from [26]

Max: maximum; min: minimum; NA: not applicable.

^a^ Unless otherwise specified.

## Transmission dynamics

**Figure 4:**
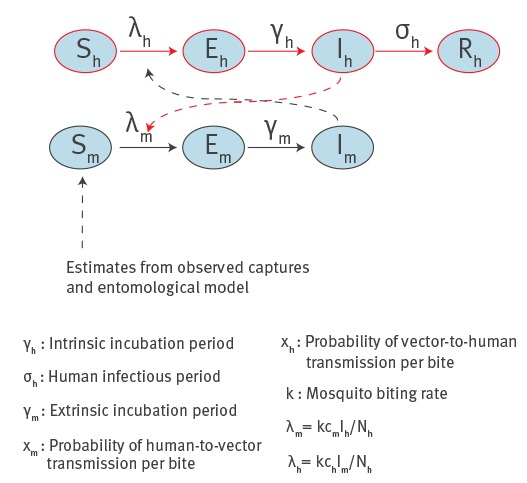
Schematic representation of the model used to estimate chikungunya transmission, Lazio region, Italy, 2017

The probability of a CHIKV outbreak, the number of symptomatic and asymptomatic cases and the daily number of notified cases at different sites were computed using a previously published stochastic transmission model [5] (Figure 4) simulated over an area of radius 300 m (i.e. ca 28 ha), according to mosquito abundance data [6], epidemiological data [10] and mosquitoes flight range [15]. Potential delays between symptom onset and notification were also accounted for (Table). A set of 10,000 model simulations was run for each site by sampling epidemiological parameters from known distributions and considering a single imported case at different times within the 1 May–15 November time window (Table). In order to predict the time of virus introduction, the symptom onset for the first notified case was considered to have occurred between 23 and 29 June in coastal sites (first recorded symptoms in Anzio: 26 June [2]) and between 12 and 18 July in urban sites (first recorded symptoms in Rome: 15 July [2]). The likely time of virus introduction was identified by selecting simulations with compliant symptom onsets.

E: exposed; I: infectious; λ: force of infection, i.e. the probability per unit of time for a susceptible to become infected; N: total population; R: recovered; S: susceptibles.

Subscripts *h* and *m* refer to humans and mosquitoes respectively. Human cases are notified with probability *p_s_p_n_*, which represent the probability of developing clinical symptoms and the probability of being detected respectively, with a delay *d* between symptom onset and detection. Parameters values are reported in the Table.

**Figure 5:**
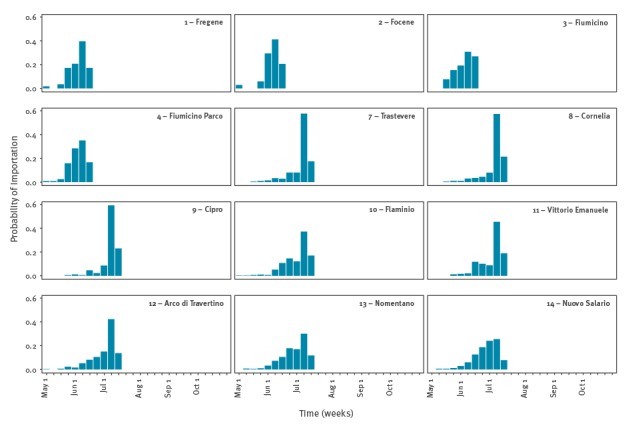
Distributions of the probable time of first chikungunya virus introduction in coastal sites (sites from 1 to 4), which were considered as representative of Anzio, and in urban sites considered as representative of Rome (sites from 7 to 14), Italy 2017

**Figure 6:**
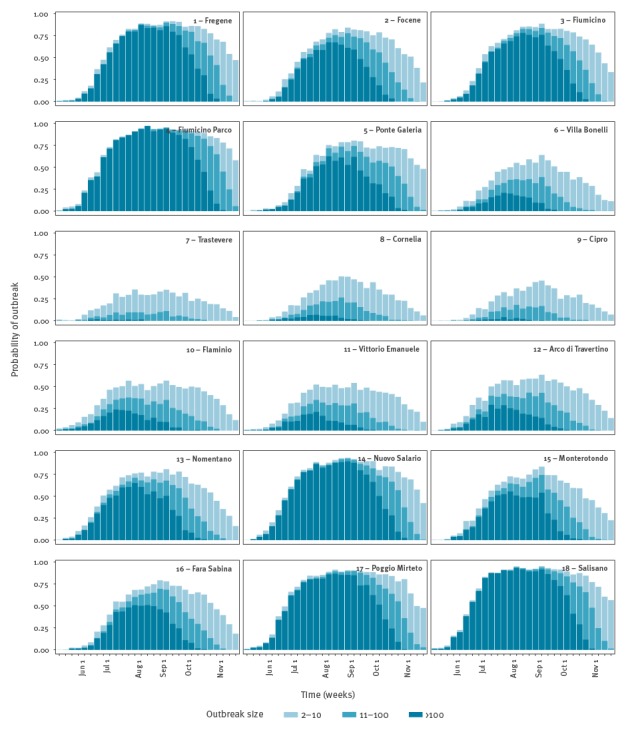
Model estimates of the probability of autochthonous transmission of chikungunya virus in 18 mosquito sampling sites in Lazio region, disaggregated by potential outbreak size, in case of a single imported case at different weeks of the year from 1 May to 15 November, Italy 2017

According to model estimates, the first CHIKV case is likely to have been imported in the first week of June in Anzio (range: 21 May–18 June, sites 1–4 in Figure 5) and in early July in Rome (range: 28 May–16 July, sites 7–14 in Figure 5). In early June the probability of occurrence of an outbreak is estimated to be higher in coastal sites (11–44%) compared with urban sites (3–34%) (Figure 6). However, in the latter sites, the probability of outbreak increases to 22–82% at the predicted time of arrival of the infection in Rome. The risk of large outbreaks is estimated to be higher in coastal and rural sites than in urban sites (Figure 6), despite the high *Ae.* *albopictus* abundance in some urban areas (Figure 2). This is explained by the higher human density in urban sites, which reduces the mosquito/human ratio and thus the risk of infection. Specifically, at the predicted time of the first case in Anzio, the number of mosquitoes per person ranged between 1.9 and 7.3 in coastal sites and between 0.4 and 2.6 in urban areas. The probability of observing additional transmission foci in unaffected areas is estimated to remain significant up to mid-November. This analysis was not performed for Guardavalle Marina due to the lack of entomological data.

Estimates do not account for the different probabilities of importation (which depend on the absolute number of infected travellers) in urban, rural and coastal sites.

## Estimates of health and economic burden

Based on observed cases that occurred before the restriction of blood donations in Lazio on 12 September [1], the estimated time of virus introduction, the notification rates (Table), the durations of infection (Table) and the available estimates on the daily blood donation rates [16], we estimated the probability that one blood sample might have been collected from an infected individual to be ca 0.73% (95% CI:  0.28–1.34%) in Anzio and 0.15% (95% CI: 0.05–0.29%) in Rome. Based on average costs and Disability Adjusted Life Years (DALY) lost per observed symptomatic CHIKV case [5], the economic burden as at 13 October is estimated at 322,000 EUR (95% CI: 222,000–477,000) with a loss of 341 DALYs (95% CI: 235–505). These estimates exclude costs related to the management of blood supplies after restrictions.

## Discussion

Our modelling estimates are subjected to uncertainties related to the actual mosquito abundance in Anzio and to the provisional nature of epidemiological data available up to now, including possible changes in the detection rates after the outbreak identification. Furthermore, the model is not suitable to evaluate the potential geographical spread of the epidemic, as it provides estimates only at the scale of 30 ha-patches, with the assumption of homogenous mixing within the patch. Critically, the high spatial heterogeneity in mosquito abundance, especially in urban areas, suggests the need to rely on information about mosquito populations at the local scale in order to assess the impact of current and future outbreaks. As shown by past surveillance records [17,18], the number of imported chikungunya cases in Lazio range from zero to seven per year, therefore suggesting that multiple importations from abroad in the city of Anzio during the summer of 2017 were unlikely; however, multiple introductions in Rome (e.g. infected tourists coming back from Anzio) are possible. This is a further possible limitation to the interpretation of results related to Rome.

Despite these limitations, the model provides relevant estimates to characterise the ongoing CHIKV outbreak in Central Italy. First, the R_0_ in Anzio is shown to be lower, but comparable to R_0_ associated with the 2007 CHIKV outbreak in Emilia Romagna and other outbreaks worldwide [3]. Second, perhaps counter-intuitively, the highest transmission potential is predicted in coastal and rural areas (due to the higher mosquito to human ratio compared with densely populated metropolitan areas), consistently with the higher incidence of CHIKV observed in Anzio compared with Rome [2]. Third, the model estimates the health and economic burden related to the outbreak, which are instrumental to evaluate cost–benefits of preventive interventions aimed to reduce mosquito vector densities. In fact, availability of information on insecticide treatments carried out after CHIKV notifications would also allow predicting their effect on mosquito population dynamics. Finally, the model predicts a risk of autochthonous transmission in Lazio region up to mid-November, as a consequence of the expected persistence of favourable climatic conditions in the area [6]. Although the number of cases is declining [19], with only 23 cases notified in October 2017, the foci of CHIKV transmission identified in the city of Latina (22 km east of Anzio) [20] and in Guardavalle Marina highlight the need to continue monitoring the outbreaks. 
